# Human Rabies — Virginia, 2017

**DOI:** 10.15585/mmwr.mm675152a2

**Published:** 2019-01-04

**Authors:** Julia Murphy, Costi D. Sifri, Rhonda Pruitt, Marcia Hornberger, Denise Bonds, Jesse Blanton, James Ellison, R. Elaine Cagnina, Kyle B. Enfield, Miriam Shiferaw, Crystal Gigante, Edgar Condori, Karen Gruszynski, Ryan M. Wallace

**Affiliations:** ^1^Virginia Department of Health; ^2^University of Virginia, Charlottesville; ^3^Piedmont Health District, Farmville, Virginia; ^4^Thomas Jefferson Health District, Charlottesville, Virginia; ^5^Division of High-Consequence Pathogens and Pathology, National Center for Emerging and Zoonotic Infectious Diseases, CDC; ^6^Lincoln Memorial University, Harrogate, Tennessee.

On May 9, 2017, the Virginia Department of Health was notified regarding a patient with suspected rabies. The patient had sustained a dog bite 6 weeks before symptom onset while traveling in India. On May 11, CDC confirmed that the patient was infected with a rabies virus that circulates in dogs in India. Despite aggressive treatment, the patient died, becoming the ninth person exposed to rabies abroad who has died from rabies in the United States since 2008. A total of 250 health care workers were assessed for exposure to the patient, 72 (29%) of whom were advised to initiate postexposure prophylaxis (PEP). The total pharmaceutical cost for PEP (rabies immunoglobulin and rabies vaccine) was approximately $235,000. International travelers should consider a pretravel consultation with travel health specialists; rabies preexposure prophylaxis is warranted for travelers who will be in rabies endemic countries for long durations, in remote areas, or who plan activities that might put them at risk for a rabies exposures.

## Case Report

On May 3, 2017, a woman aged 65 years with no preexisting health conditions began experiencing pain and paresthesia in her right arm while gardening. On May 6, the patient sought care at an urgent care facility for the arm pain. She received a diagnosis of carpal tunnel syndrome and was prescribed a nonsteroidal anti-inflammatory drug and hydrocodone. On May 7, she was evaluated at hospital A with shortness of breath, anxiety, insomnia, and difficulty swallowing water. The patient expressed concern about exposure to a toxic substance. Diagnostic test results including complete blood count, serum chemistry, D-dimer (to rule out thromboembolism), troponin, magnesium, electrocardiogram, and chest radiographs were unremarkable. She was given 0.75 mg of lorazepam for a presumed panic attack and discharged. Upon entering the car, she experienced claustrophobia and shortness of breath and immediately returned to hospital A’s emergency department (ED), where she received an additional 0.25 mg of lorazepam and was again discharged.

On May 8, she was transported from her residence by ambulance to the ED of hospital B with chest discomfort, shortness of breath, progressive paresthesia involving the right shoulder and arm, and increased anxiety. On examination, she was agitated, tachycardic, and intermittently tachypneic. Her neurologic exam was notable for dysmetria (a type of ataxia). Laboratory results were notable for elevated cardiac enzymes, a serum troponin I level of 1.05 ng/mL (normal <0.02 ng/mL), and a serum lactate level of 8.8 mmol/L (normal, 0.7–2.1 mmol/L). Electrocardiogram results* suggested acute cardiac ischemia with atypical chest pain. The patient underwent emergency cardiac catheterization, which indicated normal coronary arteries.

On the evening of May 8, the patient became progressively agitated and combative and was noted to be gasping for air when attempting to drink water. Hospital staff members questioned family about animal exposures, and the patient’s husband reported that she had been bitten on the right hand by a puppy approximately 6 weeks before symptom onset while touring in India. According to the husband, the patient cleaned the wound with the help of the tour operator but did not seek further medical treatment. The patient had no record of a pretravel health screening, did not receive rabies preexposure vaccination before the trip, nor had she ever been vaccinated against rabies.

On the morning of May 9, the patient required endotracheal intubation and mechanical ventilation for increasing somnolence, oral secretions, and oxygen desaturation; peak axillary temperature was 100.6°F (38.1°C). Electroencephalography demonstrated low-amplitude unreactive delta activity suggestive of severe encephalopathy. In light of the concern for human rabies, the patient was sedated with ketamine and midazolam, and the Virginia Department of Health was notified; because rabies PEP is ineffective for treatment of rabies and not indicated after the onset of symptoms, PEP was not administered. A lumbar puncture was performed. Cerebrospinal fluid (CSF) lactate was elevated (2.6 mmol/L; normal = 0.5–2.2 mmol/L), and CSF white blood cell count was 1 cell/*μ*L (normal = 0–5 cells/*μ*L) with 19% polymorphonuclear leukocytes and 81% mononuclear leukocytes, consistent with encephalitis. CSF, serum, saliva, and nuchal skin biopsy specimens were collected on May 9 and submitted to CDC for rabies testing on May 10.

On May 11, rabies was confirmed by the detection of rabies virus RNA by real-time reverse transcription polymerase–chain reaction (real-time RT-PCR) in saliva and skin biopsy specimens, and rabies virus antigen by direct fluorescent antibody testing of the skin biopsy (Table 1). No antirabies virus antibodies were detected in serum or CSF. Sequencing of the virus identified a canine rabies virus variant associated with dogs in India.

**TABLE 1:** Antemortem diagnostic testing* of specimens in a case of human rabies transmitted by a dog bite received in India — Virginia, 2017

Specimen type	Testing method	Date specimen collected							
		May 9	May 12	May 14	May 15	May 16	May 17	May 18	May 19
**CSF**	IFA IgG	Neg	—	Neg	—	—	Neg	Neg	—
	IFA IgM	Neg	—	Neg	—	—	Neg	Neg	—
	RFFIT	Neg	—	Neg	—	—	Neg	Neg	—
**Serum**	IFA IgG	Neg	Neg	—	Neg	Neg	Neg	—	—
	IFA IgM	Neg	Neg	—	Neg	Neg	Neg	—	—
	RFFIT	Neg	Neg	—	Neg	Neg	Neg	—	—
**Saliva**	Isolation in MNA	Neg	—	—	—	Pos	Pos	Pos	Pos
	real-time RT-PCR^†^	Pos	Pos	Pos	Pos	Pos	Pos	Pos	Pos
**Skin biopsy**	DFA	Pos	—	—	—	—	—	—	—
	real-time RT-PCR^†^	Pos	—	—	—	—	—	—	—

**Abbreviations:** CSF = cerebrospinal fluid; DFA = direct fluorescent antibody; IFA = indirect fluorescent antibody; IgG = immunoglobulin G; IgM = immunoglobulin M; MNA = mouse neuroblastoma cell culture; Neg = negative; Pos = positive; RFFIT = rapid fluorescent foci inhibition test; RT-PCR = reverse transcription–polymerase chain reaction.

* Positive result indicates detection of rabies virus antigen; negative result indicates no detection of antibody to rabies virus.

^†^ RT-PCR conducted in triplicate.

On May 13, the full Milwaukee protocol (an experimental protocol for persons with rabies that has demonstrated inconsistent, rare success) (*1*) was implemented with the addition of favipiravir (*2*). On May 15, the patient developed profuse oral secretions. On May 17, aggressive titering of ketamine and midazolam was initiated to address increased agitation, and dexmedetomidine was started to limit sympathetic responses during weaning. On May 18, repeat CSF studies continued to demonstrate no white blood cells, normal protein level of 36.0 mg/dL, and a normalized lactate level of 2.2 mmol/L. Interferon beta was started May 18 in the hope of stimulating an immune response; however, repeat CSF analysis demonstrated no evidence of antirabies virus antibodies (Table 1). Rabies virus nucleic acid was again detected in saliva by real-time RT-PCR on May 19. On May 21, the family decided to withdraw advanced medical support, and the patient died shortly thereafter. Rabies virus was isolated from brain tissue postmortem.

## Public Health Investigation

On May 9, 2017, the Thomas Jefferson Health District (TJHD) (the health district local to hospitals A and B and the urgent care center visited by the patient) initiated a local public health investigation. The district used an existing survey tool to assess exposure risk and assisted in implementing the Advisory Committee on Immunization Practices (ACIP) recommendations for PEP based on exposure risk (*3*). Hospital A infection-prevention staff members identified 18 employees who had cared for the patient, two of whom did not respond to a request for an interview. TJHD identified 240 health care providers from the urgent care center (four), emergency medical services providers (five), hospital B (223), the funeral home (seven), and the Office of the Chief Medical Examiner (one). Six employees of hospital B did not respond to interview requests. Among the 258 employees identified by TJHD and hospital A for rabies exposure risk assessments, 250 were located and assessed; rabies PEP was recommended for 72 (29%) (Figure).

**FIGURE:**
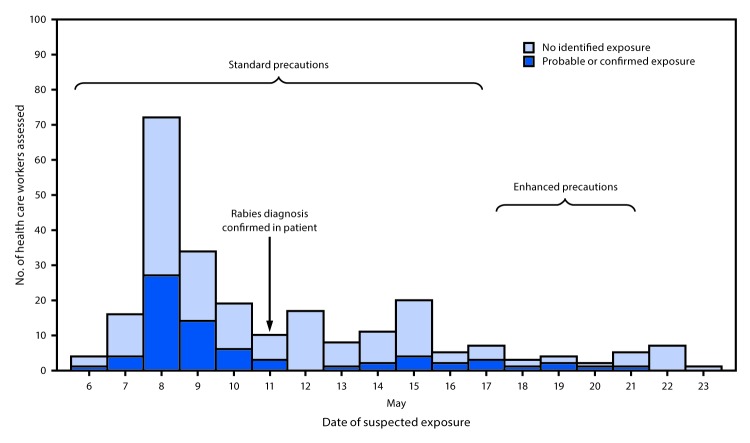
Suspected and probable or confirmed rabies virus exposures among health care workers and type of precautions implemented — Virginia, 2017* * Guidelines for precautions are available online (https://www.cdc.gov/infectioncontrol/guidelines/isolation/appendix/standard-precautions.html). Enhanced precautions were implemented in response to the patient’s urinary tract infection.

In accordance with ACIP recommendations, during May 8–10 (before the confirmed rabies diagnosis), staff members at hospital B had been recommended to follow standard infection prevention precautions (*3*). PEP was recommended for 47 health care staff members who cared for the patient during this time because of likely exposure to saliva (15.7 exposures per day) (Table 2). PEP was recommended for 15 health care workers who cared for the patient after rabies was diagnosed on May 11, but before additional precautions were implemented on May 18 (2.1 exposures per day). Implementation of enhanced contact precaution (droplet and contact precautions) during May 18–May 21 after the patient developed an antibiotic-resistant urinary tract infection presented an opportunity to assess the impact of enhanced precautions on reported exposures; PEP was recommended for five additional health care workers who cared for the patient during this period (1.3 confirmed exposures per day). The rate of daily PEP recommendations decreased significantly after the diagnosis of rabies was made (95% confidence interval [CI] = 4.2–13.5, p<0.001) but did not significantly change after enhanced precautions were implemented (rate ratio = 1.7, 95% CI = 0.6–5.3) (Figure).

**TABLE 2:** Health care worker (HCW) exposures to rabies virus while caring for a patient with rabies during three safety precaution recommendation periods — Virginia, 2017

Period	Rabies diagnosis status	Health care precautions	No. of HCW assessed	Average no. of HCW assessed per day (95% CI*)	No. (%) of HCW exposed	Average no. of HCW exposed per day (95% CI*)
May 8–10	Suspected	Standard	125	41.7 (34.8–49.5)	47 (38)	15.7 (11.6–20.7)
May 11–17	Confirmed	Standard	78	11.1 (8.9–13.8)	15 (19)	2.1 (1.2–3.5)
May 18–21	Confirmed	Enhanced^†^	14	3.5 (2.0–5.7)	5 (36)	1.3 (0.5–2.8)

**Abbreviation:** CI = confidence interval.

* Confidence intervals calculated using the Mid-P exact test with Miettinen's (1974d) modification (Rothman KJ, Boice JD. Epidemiologic analysis with a programmable calculator. Bethesda, MD: National Institutes of Health 1979).

^†^ Enhanced precautions included both droplet and contact precautions and were implemented after the patient developed an antibiotic resistant urinary tract infection.

Rabies PEP was offered to all 72 health care providers who met the ACIP definition of an exposure (*3*); eight persons declined PEP. The total pharmaceutical cost for PEP (rabies immunoglobulin and rabies vaccine) was approximately $235,000, with the cost borne by both hospitals and the local health department.

The patient’s communicability period was presumed to have begun 2 weeks before symptom onset, on April 19. The patient was a resident of a communal living facility. The Piedmont Health District interviewed 13 residents of the commune who reported close contact with the patient, four of whom met the exposure criteria: three persons had direct contact with the patient’s saliva, and one person was bitten by the patient. All four were advised to initiate PEP.

The patient had participated in a lengthy organized yoga retreat tour of India during January 28–April 5, 2017. Seventeen tour members (including the patient) from five states (California, Illinois, Maryland, North Carolina, and Virginia) and two countries (United States and Spain) and six staff members from two countries (United States and India) participated in the tour. Tour members confirmed that the patient was bitten by a puppy outside her hotel in Rishikesh, India, and that the wound was washed with water, but no further treatment was administered. Three tour members in addition to the patient reported direct contact with the same puppy; two were determined not to have been exposed to infectious materials. One, a North Carolina resident, reported having been bitten on the leg; TJHD recommended PEP for this person. A tour manual was provided to all members before travel that recommended consulting with a physician regarding any pretravel health concerns, but did not list specific health risks or pretravel vaccination recommendations. The World Health Organization International Health Regulations focal point with the Indian Ministry of Health was notified of the case, and local health authorities conducted an investigation (*4*). One rabid dog was reported from the area within the preceding 6 months, but no additional information regarding the puppy or its owner was available.

## Discussion

The canine rabies virus variant was eliminated from the United States in 2004, but remains endemic in 122 countries and is the leading global cause of human deaths secondary to zoonotic pathogens (estimated at 59,000 per year) (*5*,*6*). Recognizing that the reduced burden of human rabies deaths in the United States might result in a lack of awareness of risk when traveling abroad, CDC publishes pretravel vaccination recommendations (https://wwwnc.cdc.gov/travel). Travelers to India, which has the world’s largest incidence of dog-mediated human rabies deaths, are recommended to receive pretravel rabies vaccination if they will be involved in outdoor activities (such as camping, hiking, biking, adventure travel, and caving) that put them at risk for animal bites. In the case of the yoga retreat tour, given the extended length of the tour and the rural and community activities involved, pretravel rabies vaccination should have been considered. In the event of a suspected rabies exposure, PEP is recommended as soon as possible and has been shown to be highly effective at preventing rabies if administered prior to symptom onset (typically 3 weeks to 3 months after exposure). Persons with a history of vaccination should receive a 2-dose booster vaccination series if exposed, whereas persons with no history of vaccination require a 4-dose vaccination series with rabies immune globulin administered at the site of exposure.

CDC recommends using standard precautions when providing care to persons suspected of having clinical rabies, including wearing gowns, goggles, masks, and gloves, particularly during procedures that might result in splashes or sprays from body fluids. Enhanced precautions such as droplet and contact precautions are not considered necessary for prevention of health care–associated rabies virus exposures (https://www.cdc.gov/infectioncontrol/guidelines/isolation/appendix/standard-precautions.html) (*3*). In the case described, implementation of enhanced precautions after the patient developed a urinary tract infection did not significantly reduce the daily rate of health care worker exposures, which supports ACIP guidance that standard precautions, when applied appropriately, are adequate to minimize health care–associated rabies virus exposures. Health care–associated rabies virus exposures declined significantly after a diagnosis of rabies was confirmed, suggesting that early consideration of rabies virus infection coupled with timely diagnosis might result in improved adherence to standard infection control precautions and a reduction in exposures and related PEP costs.

This was the ninth death in the United States from rabies infection acquired while traveling or working abroad since 2008 (*7*–*10*). These events underscore the importance of obtaining a thorough pretravel health consultation, particularly when visiting countries with high incidence of emerging or zoonotic pathogens, to ensure awareness of health risks and appropriate pretravel and postexposure health care actions.

**Summary**

**What is already known about this topic?**

Canine rabies was eliminated from the United States in 2004, but remains endemic in 122 countries. Since 2008, nine persons have died from rabies in the United States following a rabies exposure abroad.

**What is added by this report?**

A U.S. citizen was bitten by a puppy while in India; rabies postexposure prophylaxis was not sought. The traveler developed rabies upon return to the United States and died during hospitalization. Seventy-two health care providers were exposed to infectious materials. Treatment for exposures cost approximately $235,000.

**What are the implications for public health practice?**

This case highlights the importance of prompt rabies diagnosis to minimize health care–associated exposures. Persons traveling internationally should seek pretravel guidance, including recommended vaccination and prophylactic measures.

## Footnotes

* The results showed 1 mm of ST segment elevation in leads AVR, V1 and V2, and 1 mm of ST segment depression in lead II, avF, and V3–V6.
